# Prediction of numbers of the accumulative confirmed patients (NACP) and the plateau phase of 2019-nCoV in China

**DOI:** 10.1007/s11571-020-09588-4

**Published:** 2020-04-27

**Authors:** Lijun Pei

**Affiliations:** School of Mathematics and Statistics, Zhengzhou University, Zhengzhou, 450001 Henan China

**Keywords:** 2019-nCoV, Prediction, Numbers of the accumulative confirmed patients (NACP), Plateau phase, Inflection points (IPs)

## Abstract

In the present study, I propose a novel fitting method to describe the outbreak of 2019-nCoV in China. The fitted data were selected carefully from the non-Hubei part and Hubei Province of China respectively. For the non-Hubei part, the time period of data collection corresponds from the beginning of the policy of isolation to present day. But for Hubei Province, the subjects of Wuhan City and Hubei Province were included from the time of admission to the Huoshenshan Hospital to present day in order to ensure that all or the majority of the confirmed and suspected patients were collected for diagnosis and treatment. The employed basic functions for fitting are the hyperbolic tangent functions $\tanh (.)$ since in these cases the 2019-nCoV is just an epidemic. Subsequently, the 2019-nCoV will initially expand rapidly and tend to disappear. Therefore, the numbers of the accumulative confirmed patients in different cities, provinces and geographical regions will initially increase rapidly and subsequently stabilize to a plateau phase. The selection of the basic functions for fitting is crucial. In the present study, I found that the hyperbolic tangent functions $\tanh (.)$ could satisfy the aforementioned properties. By this novel method, I can obtain two significant results. They base on the conditions that the rigorous isolation policy is executed continually. Initially, I can predict the numbers very accurately of the cumulative confirmed patients in different cities, provinces and parts in China, notably, in Wuhan City with the smallest relative error estimated to $0.021\%$, in Hubei Province with the smallest relative error estimated to $0.012\%$ and in the non-Hubei part of China with the smallest relative error of $-$ 0.195% in the short-term period of infection. In addition, perhaps I can predict the times when the plateau phases will occur respectively in different regions in the long-term period of infection. Generally for the non-Hubei part of China, the plateau phase of the outbreak of the 2019-nCoV will be expected this March or at the end of this February. In the non-Hubei region of China it is expected that the epidemic will cease on the 30th of March 2020 and following this date no new confirmed patient will be expected. The predictions of the time of Inflection Points and maximum NACP for some important regions may be also obtained. A specific plan for the prevention measures of the 2019-nCoV outbreak must be implemented. This will involve the present returning to work and resuming production in China. Based on the presented results, I suggest that the rigorous isolation policy by the government should be executed regularly during daily life and work duties. Moreover, as many as possible the confirmed and suspected cases should be collected to diagnose or treat.

## Introduction

It has been suggested that coronaviruses are threats to human life. This type of viruses, which was discovered and characterized in 1965, is broadly distributed in mammals and birds. In humans, the majority of the coronaviruses cause only mild respiratory infections and a limited number, such as the “Severe Acute Respiratory Syndrome” (SARS) in China (Liu et al. 2004) and the “Middle East Respiratory Syndrome” (MERS) in Saudi Arabia and South Korea, have caused more than 10,000 cumulative infected cases in the past 2 decades. Although several coronaviruses have been identified and characterized, additional unknown coronaviruses that are potential threats are to be discovered.

In December 2019, pneumonia cases of unknown reasons emerged in Wuhan, the capital of Hubei province and one of the largest cities in the central part of China. Although the majority of them were cured, they led to respiratory failures and a few patient fatalities. This outbreak of pneumonia attracted significant attention in the world. The causative agent identified by the Chinese authorities was designated 2019 novel coronavirus (2019-nCoV) by the World Heath Organization (WHO) on January 10 2020. On January 20 2020, the Chinese government classified the novel 2019-nCoV as a class A agent. A series of non-pharmaceutical interventions were implemented, namely, isolation of symptomatic persons, strict restriction of travel in Hubei province and shutdown of the public transport in various cities. Although the number of the accumulative confirmed patients (NACP) in the non-Hubei Chinese regions have decreased continually for 7 days, the effectiveness and efficiency of these interventions is questionable. In addition, when the viral infection reaches its plateau phases several factors are affected including financial costs and work abstention. Therefore, it is very crucial to reduce the outbreak of the 2019-nCoV. So far, there are nearly 20,000 confirmed cases in Wuhan and more than 40,000 confirmed cases in China, whereas several exported cases have been confirmed in other countries including Japan, South Korea, Singapore, USA, Canada, Germany, France, UK and Spain. Mathematical models were employed to investigate the viral outbreak and interesting results were obtained. The mathematical modeling of the 2019 n-CoV outbreak has been previously investigated (Chen et al. 2020; Tang et al. 2020; Rabajante 2020; Imai et al. 2020; Liu et al. 2020; Fanelli and Piazza 2020; Peng et al. 2020; Toda 2020; Sameni 2020). The reason of the outbreak has bee reported in previous studies (Chu et al. 2020; Khan et al. 2020a, b). Its clinical characteristics and laboratory test results were also studied (Qian et al. 2020; World Health Organization 2020). Its treatment and prognosis were presented in two recent studies (Chen and Du 2020; Chai et al. 2020). Its containment strategy was discussed in (Bittihn and Golestanian 2020; Hu et al. 2020). The prediction of the tendency of 2019-nCoV and notably of the NACP and of the plateau phase is of great importance at present. These goals were achieved by fitting the data of the NACP in these regions. The 2019-nCoV outbreak could not be modeled accurately due to the weak knowledge of the reasons, transmission mechanisms, effect of control policies, treatments strategies and damages. The mechanism of 2019-nCoV infection is very unclear and was studied by several scientists. However, the data contains its much information and can disclose its many natures. Therefore, these data were fitted in order to conduct the outbreak prediction. Two significant features were stated in my novel fitting method as follows:

- The first novel idea is the choice of the data of the NACP in different regions. For the China regions outside Hubei Province, i.e., the non-Hubei part, the medical conditions are sufficient and the isolation policy is well executed. All the confirmed patients can be collected and receive treatment, and the suspected cases can be collected for diagnosis and further treatment. Therefore, the outbreak of the 2019-nCoV is just a general epidemic. For Hubei Province, which includes the Wuhan City, several cabin hospitals and the Leishenshan hospital were employed following initial completeness of the Huoshenshan Hospital. The majority of the confirmed patients can be collected for treatment and most suspected patients can also be collected for diagnosis and subsequent treatment. Therefore, the outbreak of the 2019-nCoV in Wuhan City and Hubei Province are considered as a general epidemic and can be treated as such. The data were collected from the non-Hubei part of China from approximately January 20 2020, i.e., from the beginning of the policy of isolation to the present day. The data were collected from Hubei Province and from Wuhan City from February 6 2020, i.e., from the date of the initial establishment of the Huoshenshan hospital, to the present date. The data can be fitted to predict the NACP in the short-term duration and predict the initiation of the plateau phases.
- The selection of the basic functions for the fitting model is crucial for the success of the prediction. Since in both the above cases, the 2019-nCoV is just an epidemic, this suggests that it will initially spread rapidly and subsequently exhibit a tendency to disappear. Therefore, the numbers of the cumulative confirmed patients in different cities, provinces and geographical locations will initially increase rapidly and subsequently remain constant when reaching the plateau phase of the viral infection. In the present study, the hyperbolic tangent functions $\tanh (.)$ were used that can satisfy the aforementioned conditions. Therefore, the hyperbolic tangent function $\tanh (.)$ was set as the basic function for fitting. It laid the foundation of the success of the fitting model and further enhanced the prediction success of the 2019-nCoV infection.

By this novel method, two significant results were obtained based on the conditions that the rigorous isolation policy is executed continually. Initially, the numbers of the accumulative confirmed patients in different cities, provinces and geographical locations in China were predicted very accurately in the short term period of infection. Moreover, the times of the plateau phases were determined in different places in the long-term period of infection. Generally, in the non-Hubei China part, the NACP of 2019-nCoV will tend to constant from approximately February 23 2020 and its maximum infectivity will be theoretically achieved by March 30 2020. Following this date, no additional infected patient will be expected to be diagnosed. Based on the present results, it is suggested that the rigorous isolation policy by the government should be executed continually.

The remaining part of this article is organized as follows: In Sect. 2, the novel fitting method of the outbreak of 2019-nCoV in China is proposed, and the selection of the data and basic functions is presented. The validation of this novel method was achieved in the data derived from the SARS infection in 2003 in China Mainland and Hongkong, which are presented in Sect. 3. The results of the prediction of NACP and of the plateau phase, as well as of the IPs of 2019-nCoV in China are presented in Sect. 4. Finally, I present some concluding remarks in Sect. 5.

## The novel fitting method

Initially, I will present the novel fitting method for the prediction of NACP and the plateau phase of 2019-nCoV in China. The success of the fitting or prediction depends on the selection of the data, the basic functions for this fitting and the fitting method. I will describe all three components in this section.

### Data of the NACP

The data of the NACP must correspond to the epidemic characteristics. They must fit into the epidemic pattern. All the confirmed patients must be collected for treatment and the suspected patients can be collected for diagnosis. The effective treatment of the infected patients and the efficient diagnosis of the suspected cases should be ensured. This is the basis of the principle to which the government adheres regarding as many as possible the confirmed and suspected should be collected to diagnose and treat. With regard to the Chinese regions outside of Hubei Province, i.e., the non-Hubei part of China, the medical conditions are sufficient to treat the infected cases and ultimately contain the spread of the virus. Therefore the confirmed patients can be collected for the appropriate treatment and the suspected patients can be collected for diagnosis and further treatment, so that the outbreak of the 2019-nCoV in this part will be considered as a general epidemic. Therefore the data from the 21st of January 2020, which was the beginning of the strict isolation policy in the non-Hubei region were used for fitting. The data of the NACP in cities and provinces with major viral outbreak are presented in Tables 1 and 2. All data are collected from the official websites of the Health Commissions in these regions.

**Table 1:** NACP in the serious outbreak Non-Hubei cities and provinces. I

Regions	1.20	1.21	1.22	1.23	1.24	1.25	1.26	1.27	1.28
Non-Hubei	N	N	N	N	N	923	1321	1801	2420
Beijing	N	10	14	26	36	54	68	91	102
Shanghai	1	9	16	20	33	40	53	66	80
Zhengzhou	N	1	2	3	6	20	29	37	40
Xinyang	N	N	N	1	5	22	23	29	32
Nanyang	N	N	N	N	8	15	19	26	31
Zhoukou	N	N	N	1	4	5	11	15	19
Gansu	N	N	N	2	4	7	14	19	24
Lanzhou	N	N	N	N	1	4	8	11	14

**Table 2:** NACP in the serious outbreak Non-Hubei cities and provinces. II

Regions	1.29	1.30	1.31	2.1	2.2	2.3	2.4	2.5	2.6	2.7
Non-Hubei	3125	3886	4638	5306	6028	6916	7646	8353	9049	9593
Beijing	114	132	156	183	212	228	253	274	297	315
Shanghai	101	128	153	177	193	208	233	254	269	281
Zhengzhou	46	50	56	65	72	85	92	102	112	120
Xinyang	42	49	70	88	99	112	138	164	176	192
Nanyang	51	61	66	76	84	99	107	111	118	128
Zhoukou	25	36	38	40	47	52	56	59	60	62
Gansu	26	29	35	40	51	55	57	62	67	71
Lanzhou	14	15	20	23	24	26	27	27	27	32

The Hubei Province, which includes Wuhan City, was thoroughly assessed. Following the establishment of the Huoshenshan hospital, the cases reported in several cabin hospitals and subsequently in the Leishenshan Hospital were examined. The majority of the confirmed patients were collected for successful treatment, and most of the suspected patients were collected for diagnosis and further treatment. Therefore, the outbreak of the 2019-nCoV in Wuhan City and even Hubei Province is considered a general epidemic, suggesting that it should be treated as such. The data from Wuhan City and Hubei Province were collected from the establishment of the Huoshenshan hospital to the present date to ensure that all or the majority of the confirmed and suspected cases could be collected for diagnosis and treatment. The data of the NACP in Wuhan and Hubei Province are presented in Table 3. All the data were collected from the official websites of the Health Commissions in Hubei Province and Wuhan City.

A notable change was noted on February 12 2020. The numbers of the cumulative clinical confirmed patients were also added to those of the cumulative confirmed patients in Hubei Province and in Wuhan City. More than 10,000 cases of this type were added into the data collected by February the 12th. Therefore, the present data are differentiated from the restrictive confirmed standards to the relaxed confirmed standards. However, the data were collected based on the old restrictive confirmed standards. In future studies, the data should be fitted to the new relaxed confirmed standards of Hubei Province and Wuhan City in order to predict the number of the accumulative confirmed patients and the plateau phase of the 2019-nCoV infection. These data are subsequently fitted to the following novel basic functions.

**Table 3:** NACP in Wuhan City and Hubei Province

Regions	2.6	2.7	2.8	2.9
Hubei	22,112	24,953	27,100	29,631
Wuhan	11,618	13,603	14,982	16,902

I will fit these data by the following novel basic functions.

### Basic functions for this fitting

Usually, the power functions $1, x, x^{2}, x^{3}, x^{4}, \ldots$ are used as the basic functions for fitting the data. However, the data of the infectious diseases require a different set of basic functions. Despite fitting of the data, a usual fluctuation may be noted and consequently the tendency-like plateau phase can not be predicted. Therefore, the selection of the basic functions for this fitting is crucial. The infectious diseases are characterized by the numbers of the cumulative confirmed patients. They initially increase rapidly and finally stabilize. At that time period no confirmed patient presents. Following isolation and treatment, the majority of the confirmed patients will recover and a limited number will not survive the infection. At last, the infectious diseases are controlled and eradicated. It is well known that the hyperbolic tangent functions $\tanh (.)$ exhibit two properties: an initial rapid increase and a final phase with constant. Based on these two properties, the functions are rearranged to: $1, \tanh (0.1 x), \tanh (0.2 x), \tanh (0.3 x), \ldots$ or $1, \tanh (0.2 x), \tanh (0.4 x), \tanh (0.6 x), \ldots$ as the basic functions for fitting the data of the NACP in these regions. The accuracy of the fitting by this novel method is excellent since not only it can predict the NACP in these regions in the next day with very small relative errors, but it can also plot the evolution curves of the NACP in the long-run period of infection and perhaps it can be used to estimate the days when the plateau phase comes.

### Fitting method

Initially, the novel fitting method was used for estimation of the NACP. The number of infected cases per day can be predicted in some regions by the novel fitting method and the fitting function. A plot can be constructed and the days required for the plateau phase can be estimated. For example, in Nanyang City, a serious outbreak city that is close to Wuhan City in the Henan Province, the restrictive isolation was executed from January 25 to February 7 (Tables 1, 2). The data were fitted using the following basic functions: $1, \tanh (0.1 x), \tanh (0.2 x), \tanh (0.3 x), \ldots$. The equation was rearranged as follows,

$$f(x)=6.4056 + 308.92 \tanh (0.1 x) - 261.789 \tanh (0.2 x) + 164.811 \tanh (0.3 x) - 65.3425 \tanh (0.4 x).$$

The number of cases on February 8 2020 was $f(16) = 129.653 \approx 130$. The actual number was 133 and the relative error was $-2.26\%$. The fitting results are shown in Fig. 6a. The fitting was optimal. The prediction, which is described by the evolution curve is displayed in Fig. 6b. Obviously it will be constant from February 22 2020. Shanghai City is a very important international city in China and the NACP from January 20 to February 7 (Tables 1, 2) following execution of the restrictive isolation could be fitted in the basic functions: $1, \tanh (0.1 x), \tanh (0.2 x), \tanh (0.3 x), \ldots$. Its expression was as follows,

$$f(x)= 86.6827 + 1597.22 \tanh (0.1 x) - 5473.09 \tanh (0.2 x) + 14560.9\tanh (0.3 x) - 24,272. \tanh (0.4 x) + 21,611.7 \tanh (0.5 x) - 7767.27 \tanh (0.6 x).$$

The number estimated in February 8, 2020 was $f(20) = 290.174 \approx 290$. The actual number was 292 and the relative error was $-0.685\%$. The fitting result is shown in Fig. 3a. The fitting was optimal. The prediction that contained the evolution curve is displayed in Fig. 3b. It is expected that it will tend to be constant from approximately February 20, 2020. Another example can be obtained for the non-Hubei region of China part. The NACP in that region was reported from January 25 to February 7 (Tables 1, 2), when the strict isolation was executed. The data could be fitted with the basic functions as follows: $1, \tanh (0.1 x), \tanh (0.2 x), \tanh (0.3 x), \ldots$. The following formula was obtained,

$$f(x)=959.537 + 35049.5 \tanh (0.1 x) - 77153.2 \tanh (0.2 x) + 169568. \tanh (0.3 x) - 243464. \tanh (0.4 x) + 187331. \tanh (0.5 x) - 59145.8 \tanh (0.6 x).$$

The number estimated on February 8 2020 was $f(15) = 10162.9 \approx 10,163$. The actual number was 10,098, and the relative error was $0.64\%$. The fitting result is shown in Fig. 8a. The fitting was excellent. The prediction, i.e., the evolution curve is displayed in Fig. 8b. It is expected for this curve to stabilize from the 23th of February 2020. All fittings and the evolution curves for the different regions are presented in Figs. 2, 3, 4, 5, 6, 7, 8, 9 and 10.

With regard to the prediction of the next day, i.e. on the 9th of February 2020, the method is similar. The actual number on the 8th of February 2020 was added into the old data and fitted with the new method and the above basic functions. The fitting functions could be obtained and the number of infected cases on February 9 2020 could also be obtained. The fitting results and the evolution curves were obtained. The prediction of NACP in the continuous days is presented in Tables 4 and 5.

## Validation of the novel fitting method in data of SARS in China mainland and Hongkong

**Fig. 1:**
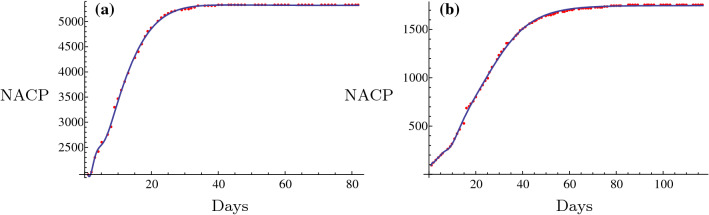
Fitting and prediction of SARS in China Mainland and Hongkong in 2003 respectively in **a** and **b**

Initially, this novel fitting method was employed to the fitting and prediction of the data of SARS in China Mainland and Hongkong in 2003 to assess the effectiveness of this method. The results are presented in Fig. 1. Apparently, the fitting was excellent and could predict the evolution of SARS in China Mainland and in Hongkong in 2003 qualitatively and quantitatively. The data of SARS in China Mainland were obtained from the official website of WHO (World Health Organization) from April 21 2003 to July 11 2003 (https://www.who.int/csr/sars/country/en/). The data from the Hongkong were obtained from the website from the March 17 2003 to the 1st of July 2003 (https://www.who.int/csr/sars/country/en/).

The data implied that the novel fitting method is excellent for SARS in 2003 and perhaps valid and effective for the 2019-nCoV. In the next section, this fitting method was employed to the prediction of NACP and the plateau phase of 2019-nCoV in China.

## Fitting results

### Prediction of NACP in different regions of China

In this subsection, the prediction of NACP will be presented in the continuous days at different regions in Tables 4 and 5.

**Table 4:** Prediction of NACP in different regions for a continuous time period. I

Regions	2.8	2.9	2.10
Non-Hubei	10,163/10,098, 0.64%	10,583/10,542, 0.38%	10,950/10,922, 0.26%
Beijing	328/326, 0.61%	340/337, 0.89%	350/342, 2.34%
Shanghai	290/292, $-$ 0.69%	300/295, 1.7%	306/302, 1.33%
Zhengzhou	127/126, 0.79%	132/130, 1.54%	137/132, 3.79%
Xinyang	208/205, 1.46%	219/220, $-$ 0.46%	231/228, 1.32%
Nanyang	130/133, $-$ 2.26%	135/134, 0.75%	138/136, 1.47%
Zhoukou	64/62, 3.23%	65/65, 0	66/65, 1.54%
Gansu	74/79, $-$ 6.33%	79/83, $-$ 4.82%	84/86, $-$ 2.33%
Lanzhou	30/33, $-$ 9.1%	32/33, $-$ 3.03%	33/35, $-$ 5.71%
Hubei	N	N	32,136/31,728, 1.29%
Wuhan	N	N	18,838/18,454, 2.08%

**Table 5:** Prediction of NACP in different regions for a continuous time period. II

Regions	2.11	2.12	2.13
Non-Hubei	11,265/11,287, $-$ 0.2%	11,554/11,598, $-$ 0.38%	11,808/11,865, $-$ 0.48%
Beijing	356/352, 1.14%	362/366, $-$ 1.09%	370/372, $-$ 0.54%
Shanghai	311/306, 1.63%	315/313, 0.64%	319/318, 0.32%
Zhengzhou	139/137, 1.46%	142/141, 0.71%	144/142, 1.41%
Xinyang	239/231, 3.46%	243/240, 1.25%	249/243, 2.47%
Nanyang	138/138, 0	142/145, $-$ 2.07%	145/146, $-$ 0.69%
Zhoukou	67/66, 1.52%	67/68, $-$ 1.47%	68/69, $-$ 1.45%
Gansu	87/86, 1.16%	89/87, 2.3%	90/90, 0
Lanzhou	34/35, $-$ 2.86%	35/35, 0	36/35, 2.86%
Hubei	33,208/33,366, $-$ 0.47%	34,476/34,874, $-$ 1.14%	35,754/36,602, $-$ 2.32%
Wuhan	19,513/19,558, $-$ 0.23%	20,318/20,630, $-$ 1.51%	21,425/21,960, $-$ 2.44%

All fittings and the evolution curves for different regions are represented in Figs. 2, 3, 4, 5, 6, 7, 8, 9 and 10.

**Fig. 2:**
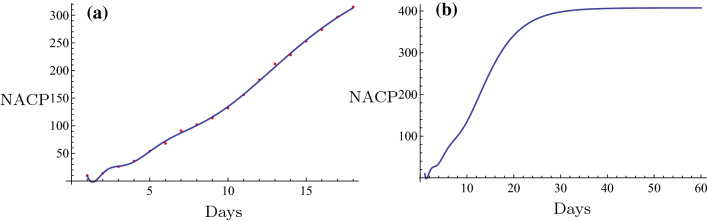
The fitting (**a**) and the evolution curve (**b**) of NACP for Beijing

**Fig. 3:**
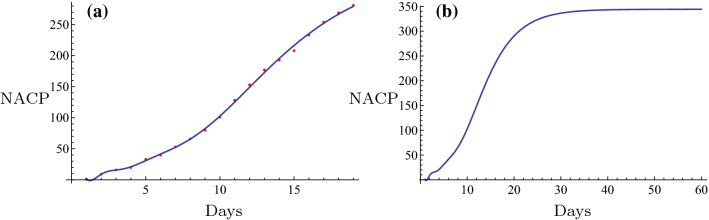
The fitting (**a**) and the evolution curve (**b**) of NACP for Shanghai

**Fig. 4:**
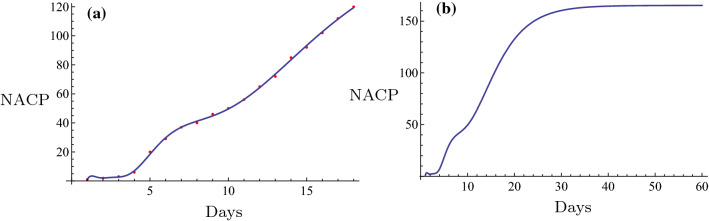
The fitting (**a**) and the evolution curve (**b**) of NACP for Zhengzhou, Henan Province

**Fig. 5:**
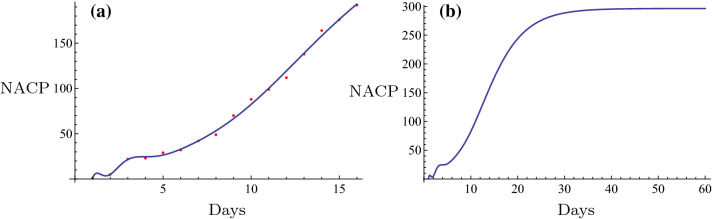
The fitting (**a**) and the evolution curve (**b**) of NACP for Xinyang, Henan Province

**Fig. 6:**
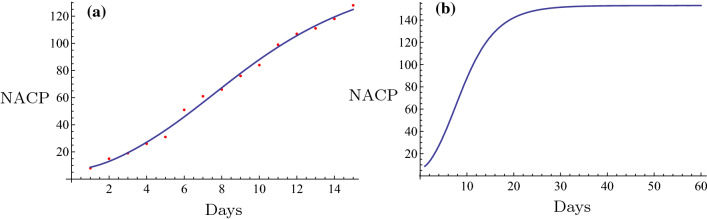
The fitting (**a**) and the evolution curve (**b**) of NACP for Nanyang, Henan Province

**Fig. 7:**
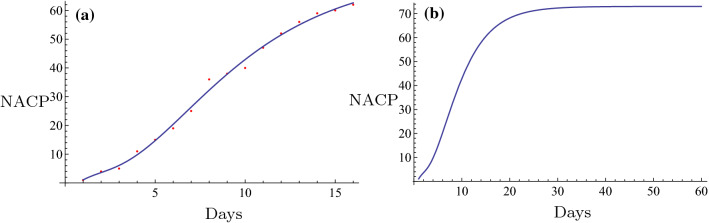
The fitting (**a**) and the evolution curve (**b**) of NACP for Zhoukou, Henan Province

**Fig. 8:**
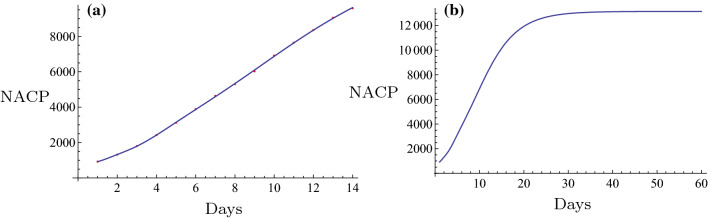
The fitting (**a**) and the evolution curve (**b**) of NACP for Non-Huibei regions

**Fig. 9:**
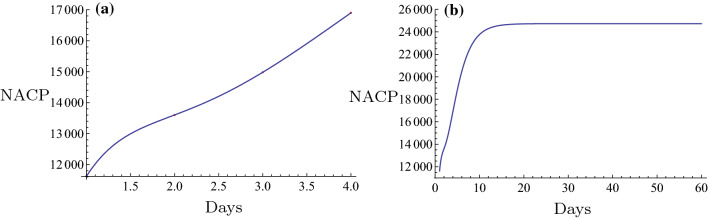
The fitting (**a**) and the evolution curve (**b**) of NACP for Wuhan, Hubei Province

**Fig. 10:**
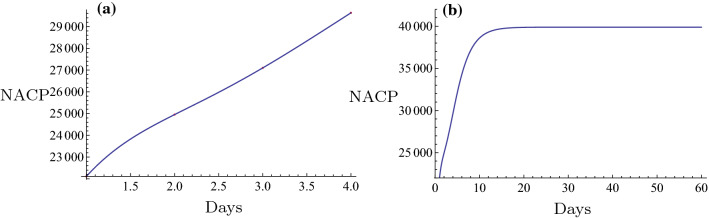
The fitting (**a**) and the evolution curve (**b**) of NACP for Hubei Province

### Prediction of inflection points in different regions of China

**Fig. 11:**
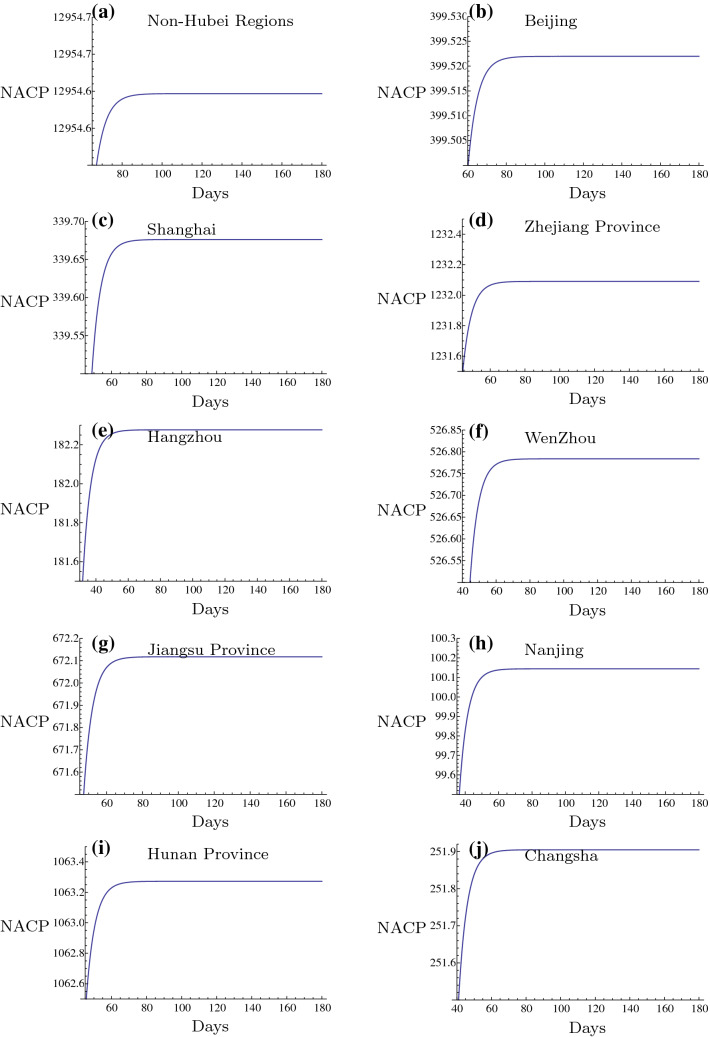
Inflection points in different regions I. The intersection points of the evolution curves with the horizontal axis are the times of the Inflection Points and the level parts mean the maximums of NACP for different regions respectively

**Fig. 12:**
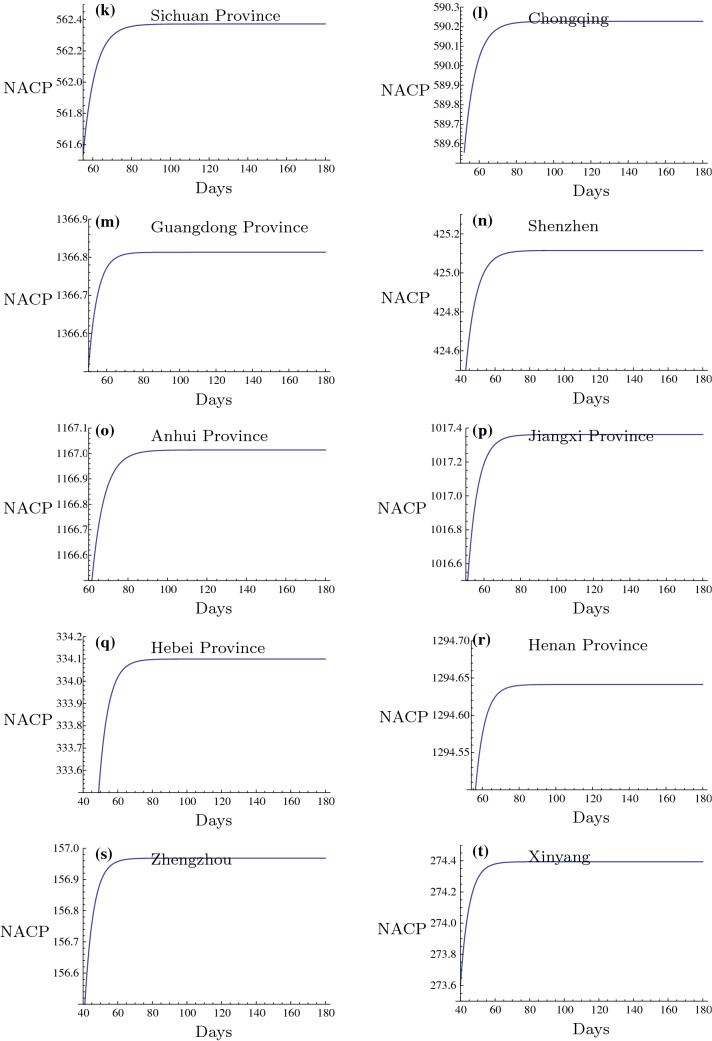
Inflection points in different regions II. The intersection points of the evolution curves with the horizontal axis are the times of the Inflection Points and the level parts mean the maximums of NACP for different regions respectively

**Fig. 13:**
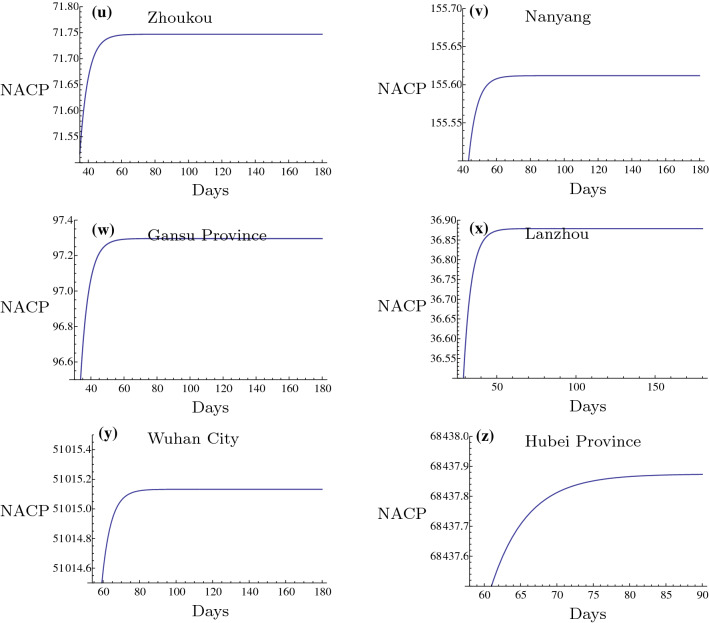
Inflection points in different regions III. The intersection points of the evolution curves with the horizontal axis are the times of the Inflection Points and the level parts mean the maximums of NACP for different regions respectively

In this subsection, the inflection points (IP) in different Chinese regions are presented. IP stands for the maximum of NACP that was achieved. After this point, the infectious disease will be controlled and the majority of the confirmed patients will recover, whereas a low number of cases will succumb to the disease. The prediction of this point is of great importance to the inhibition of the 2019-nCoV outbreak. In the present study, the prediction of IPs in the 2019-nCoV outbreak in different regions of China is presented (Figs. 11, 12, 13 and Tables 6, 7, 8, 9). These regions are usually important cities or the main affected regions around Hubei province. It is of great importance to investigate the date of the 2019-nCoV termination. It is very helpful for the prevention of 2019-nCoV outbreak to construct a plan for their daily life, returning to work and resuming production by the government. It can be deduced that the cities in the mainland China outside of Hubei Province should be able to control the outbreak of 2019-nCoV before March 30 2020 and after this date no confirmed patient should be reported. The nearby regions, especially the severe outbreak regions, such as Henan, Hunan, Jiangxi and Anhui Provinces, the important industrial and financial regions, such as Guangdong, Zhejiang, Jiangsu Provinces and the important international cities, such as Beijing and Shanghai are also predicted in Figs. 11, 12, 13 and Tables 6, 7, 8, 9. Since the medical conditions and curative efficiency are being improved, the NACP of Hubei Province and Wuhan City can not represent the real number of the infected subjects. Thus, the NACP or IP can not be predicted effectively. But in this paper, I have tried to predict the NACPs and IPs for Wuhan City and Hubei Province. The results of NACPs are excellent also and presented in Tables 10, 11 and 12. The results for IPs in these two regions are presented in Fig. 13$(y-z)$ and Table 6.

Based on the fact that the rigorous isolation policy is executed continually, the results of the present study are very significant. Firstly, the numbers of the infected subjects can be predicted very accurately leading to the cumulative number of confirmed patients in different regions of China. Notably, Hubei Province exhibited the smallest relative errors ($0.012\%$), followed by Wuhan City ($0.021\%$). In addition, the non-Hubei Chinese region exhibited larger relative errors ($-0.195\%$) in the short-term period of infection. Moreover, it is possible to predict the time points when the plateau phases are developed in different regions in the long-run period of infection. It was generally shown for the non-Hubei Chinese region that the NACP of the 2019-nCoV infection would tend to reach a constant state of growth from February 23 2020. This evidence is considered very important for the fighting against the 2019-nCoV outbreak in China.

Recently, there are increasing imported confirmed patients in the international cities such as Beijing, Shanghai, Guangzhou since more and more Chinese peoples returned back from the other countries suffering from the worse outbreak of 2019-nCoV. Since the numbers of the coming back peoples from these countries in everyday and the rates of infection in these countries are different, so the number of the imported confirmed patients are stochastic. It is not similar to the situation of China Mainland. It has no any relation to the outbreak of 2019-nCoV in China Mainland. I can not predict it accurately by the present method in this paper. I will study it in the separate paper in the future. It is a challenge of the fighting against the 2019-nCoV in China.

**Table 6:** Prediction of IP in different Chinese regions. I

Items	Non-Hubei	Beijing	Shanghai	Zhejiang	Hangzhou	Wuhan	Hubei
1st Day of Data	25 Jan.	21 Jan.	20 Jan.	24 Jan.	24 Jan.	12 Feb.	12 Feb.
Day of IP	30 Mar.	21 Mar.	8 Mar.	8 Mar.	26 Feb.	10 Apr.	12 Apr.
Max. of NACP	12,955	400	340	1232	182	51,015	68,438

**Table 7:** Prediction of IP in different Chinese regions. II

Items	Wenzhou	Jiangsu	Nanjing	Hunan	Changsha	Sichuan
1st Day of Data	24 Jan.	23 Jan.	23 Jan.	23 Jan.	23 Jan.	21 Jan.
Day of IP	7 Mar.	9 Mar.	28 Feb.	8 Mar.	3 Mar.	15 Mar.
Max. of NACP	527	672	100	1063	252	562

**Table 8:** Prediction of IP in different Chinese regions. III

Items	Chongqing	Guangdong	Shenzhen	Anhui	Jiangxi	Hebei
1st Day of Data	22 Jan.	23 Jan.	23 Jan.	21 Jan.	22 Jan.	22 Jan.
Day of IP	13 Mar.	12 Mar.	5 Mar.	22 Mar.	12 Mar.	10 Mar.
Max. of NACP	590	1367	425	1167	1017	334

**Table 9:** Prediction of IP in different Chinese regions. IV

Items	Henan	Zhengzhou	Xinyang	Zhoukou	Nanyang	Gansu	Lanzhou
1st Day of Data	22 Jan.	21 Jan.	23 Jan.	23 Jan.	24 Jan.	23 Jan.	24 Jan.
Day of IP	16 Mar.	1 Mar.	5 Mar.	25 Feb.	6 Mar.	2 Mar.	23 Feb.
Max. of NACP	1295	157	274	72	156	97	37

**Table 10:** Prediction of NACP in different regions in recent time period. I

Regions	2.24	2.25	2.26
Non-Hubei	12,961/12,872, 0.691%	12,977/12,877, 0.777%	12,988/12,901, 0.674%
Hubei	65,129/64,786, 0.529%	65,231/65,187, 0.0675%	65,507/65,596, $-$ 0.136%
Wuhan	47,011/47,071, $-$ 0.128%	47,535/47,441, 0.198%	47,927/47,824, 0.215%

**Table 11:** Prediction of NACP in different regions in recent time period. II

Regions	2.27	2.28	2.29
Non-Hubei	12,996/12,910, 0.666%	13,003/12,914, 0.689%	13,008/12,917, 0.705%
Hubei	65,850/65,914, $-$ 0.097%	66,152/66,337, $-$ 0.279%	66,496/66,907, $-$ 0.614%
Wuhan	48,280/48,137, 0.297%	48,567/48,557, 0.021%	48,889/49,122, $-$ 0.474%

**Table 12:** Prediction of NACP in different regions in recent time period. III

Regions	3.1	3.2	3.3
Non-Hubei	13,010/12,923, 0.673%	13,012/12,934, 0.603%	13,012/12,938,0.572%
Hubei	66,925/67,103, $-$ 0.265%	67,225/67,217, 0.012%	67,421/67,332, 0.132%
Wuhan	49,296/49,315, $-$ 0.0385%	49,574/49,426, 0.299%	49,752/49,540, 0.428%

Based on these results, it is suggested that not only the rigorous isolation policy by the government should be executed continually, but also the concomitant diagnosis and treatment of—as many as possible—confirmed and suspected cases should be facilitated. This will speed up the conquer of the viral outbreak.

## Conclusions

In the present study, the novel fitting method was employed to predict the NACP and the plateau phase of the 2019-nCoV infection in different regions of China. The data were collected during different time periods of infection occurring in different regions of China to ensure that as many as possible confirmed and suspected cases could be collected for diagnosis or treatment. The hyperbolic tangent functions were used as the basic functions for the fitting method. Two significant results were obtained as follows: Firstly, the NACP could be predicted very accurately in different regions of China, notably in Wuhan City and Hubei Province with very small relative errors. In the non-Hubei region of China larger relative errors were noted in the short-term period of infection. Secondly, the time point at which the plateau phases occur can be predicted in different regions in the long-run period of infection. Generally for the non-Hubei Chinese regions, the plateau phase of 2019-nCoV was noted at approximately March 30 2020 and after this time period no new confirmed patients were identified. The predictions of the time of Inflection Points (IPs) and maximum NACP for certain important regions were also presented. These measures are very important for the prevention of the 2019-nCoV outbreak and for returning to work and resuming production in their daily life and work duties in China. Based on these results, it is suggested that the rigorous isolation policy should be executed by the government continually during the daily life and that as many as possible confirmed and suspected cases should be collected for diagnosis or treatment.
